# The phenotypes of ATG9, ATG16 and ATG9/16 knock-out mutants imply autophagy-dependent and -independent functions

**DOI:** 10.1098/rsob.150008

**Published:** 2015-04-15

**Authors:** Qiuhong Xiong, Can Ünal, Jan Matthias, Michael Steinert, Ludwig Eichinger

**Affiliations:** 1 Zentrum für Biochemie, Medizinische Fakultät, Universität zu Köln, Joseph-Stelzmann-Strasse 52, Köln 50931, Germany; 2 Institut für Mikrobiologie, Technische Universität Braunschweig, Spielmannstrasse 7, Braunschweig 38106, Germany; 3 Fen Fakültesi, Türk-Alman-Üniversitesi, Istanbul 34820, Turkey; 4 Helmholtz Centre for Infection Research, Braunschweig 38124, Germany

**Keywords:** *Dictyostelium*, autophagy, development, phagocytosis, proteasome, protein aggregate

## Abstract

Macroautophagy is a highly conserved intracellular bulk degradation system of all eukaryotic cells. It is governed by a large number of autophagy proteins (ATGs) and is crucial for many cellular processes. Here, we describe the phenotypes of *Dictyostelium discoideum* ATG16^−^ and ATG9^−^/16^−^ cells and compare them to the previously reported ATG9^−^ mutant. ATG16 deficiency caused an increase in the expression of several core autophagy genes, among them *atg9* and the two *atg8* paralogues. The single and double ATG9 and ATG16 knock-out mutants had complex phenotypes and displayed severe and comparable defects in pinocytosis and phagocytosis. Uptake of *Legionella pneumophila* was reduced. In addition, ATG9^−^ and ATG16^−^ cells had dramatic defects in autophagy, development and proteasomal activity which were much more severe in the ATG9^−^/16^−^ double mutant. Mutant cells showed an increase in poly-ubiquitinated proteins and contained large ubiquitin-positive protein aggregates which partially co-localized with ATG16-GFP in ATG9^−^/16^−^ cells. The more severe autophagic, developmental and proteasomal phenotypes of ATG9^−^/16^−^ cells imply that ATG9 and ATG16 probably function in parallel in autophagy and have in addition autophagy-independent functions in further cellular processes.

## 2. Background

Macroautophagy (hereafter autophagy) is the major lysosomal route for the turnover of cytoplasmic components. It serves as a housekeeping mechanism and as a cellular response to different stresses such as starvation, the presence of protein aggregates or intracellular bacteria and is essential for temporary cell survival [1]. In addition, it also plays a key role in a range of normal developmental processes such as sporulation in *Saccharomyces cerevisiae*, fruiting body formation in *Dictyostelium discoideum*, dauer development of *Caenorhabditis elegans* and pupa formation in *Drosophila melanogaster* [2,3]. Furthermore, it may promote type II programmed cell death, a type of programmed cell death distinct from apoptosis [4,5].

The autophagy machinery is highly complex and conserved in all eukaryotes. It is composed of many core and accessory autophagy-related (ATG) proteins, many of which have been initially characterized in yeast [6]. Autophagy can be subdivided into three main stages: initiation, expansion and lysosomal degradation. Initially, upon appropriate signals, the phagophore or isolation membrane is formed. This structure is elongated and thereby enwraps cytoplasmic constituents such as macromolecules and organelles, until its edges are fused with each other to form a double-membrane structure called the autophagosome. Finally, fusion of the outer membrane of the autophagosome with the lysosome (or vacuole in yeast) leads to the formation of the autophagolysosome in which the sequestered cytoplasmic components together with the inner membrane of the autophagosome are degraded by the resident hydrolases [7–9].

**Figure 1.**
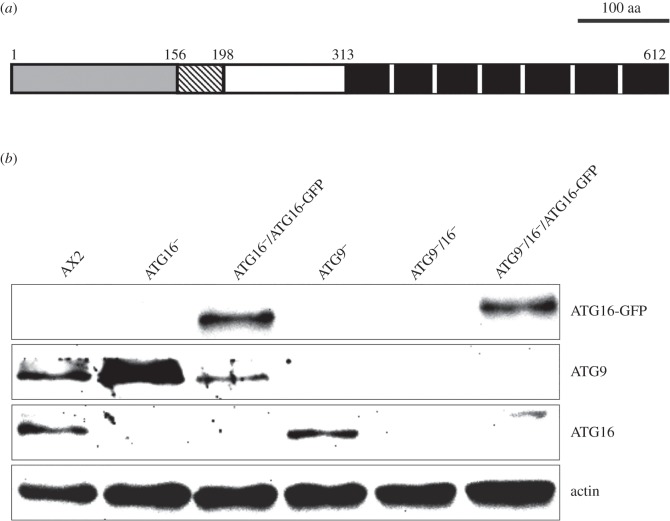
Domain structure of ATG16 and verification of generated strains by immunoblotting. (*a*) Domain structure of the 612 amino acid ATG16 protein (http://smart.embl-heidelberg.de/). The ATG5 binding site (light grey) and the coiled-coil domain responsible for homodimerization (hatched box) are located in the N-terminal half of the protein. The seven WD40 repeats make up the C-terminal half and are depicted as black rectangles. (*b*) Verification of mutant strains. Western blot of total cell lysates of wild-type AX2, the ATG16^−^, ATG9^−^ and ATG9^−^/16^−^ mutants and two mutants that express GFP fused C-terminally to ATG16 in the ATG16^−^ mutant (ATG16^−^/ATG16-GFP) or, respectively, in the ATG9^−^/16^−^ double mutant (ATG9^−^/16^−^/ATG16-GFP). Top row: anti-GFP antibody; second row from top: anti-ATG9 antibody; third row from top: anti-ATG16 antibody; bottom row: Act1-7 anti-actin antibody.

The core autophagy protein ATG16 has been shown to be involved in the expansion of the isolation membrane [10,11]. The protein is part of one of the two ubiquitin-like protein conjugation systems of the autophagy machinery [9]. It associates non-covalently with the ATG12–ATG5 conjugate and thereby forms a tetrameric complex consisting of two ATG12–ATG5 conjugates bound to an ATG16 dimer [12,13]. In this tetrameric complex, ATG16 determines the binding site at the preautophagosomal structure (PAS) in yeast or the isolation membrane in higher eukaryotes [10,14], while ATG12–ATG5 catalyses the lipidation of ATG8 with phosphatidylethanolamine (PE) through its E3-ligase activity [15]. Mammals possess two ATG16 paralogues, ATG16L1 and ATG16L2, and *D. discoideum* ATG16 is the orthologue of mammalian ATG16L1 [3]. The ATG16L1 protein is composed of an N-terminal half which harbours the ATG5 binding site, a coiled-coil domain responsible for homodimerization, and binding sites for clathrin, FIP200 and Rab33B [12,13,16–19]. The C-terminal half is composed of seven WD-repeats, which fold into a β-propeller structure, that contains binding sites for NOD 1 and 2, TMEM59 and ubiquitin. It is assumed that this domain is crucial for its role in xenophagy [20–24]. The *Dictyostelium* orthologue has a similar size and structure as mammalian ATG16L1 (figure 1*a*).

ATG16L1 deficiency in mouse embryonic fibroblasts disrupted the recruitment of the ATG12–ATG5 conjugate to the isolation membrane and resulted in a loss of ATG8 (LC3) linkage to PE [25]. Recently, a non-synonymous single-nucleotide polymorphism (A to G; T300A in the protein) was identified in the *ATG16L1* gene as a risk factor for the development of Crohn's disease (CD) [26,27]. The T300A mutation had no effect on ATG16L1 binding to ATG5 and on basal autophagy. However, the T300A variant showed impaired xenophagy against *Salmonella typhimurium* suggesting that the increased risk of CD is due to less-efficient bacterial capture by autophagy in cells expressing the mutant variant [28]. ATG9 is so far the only known integral membrane protein of the core autophagy machinery and is thought to deliver membrane lipids to the site of autophagosome formation [29–32].

*Dictyostelium discoideum* amoebae grow as separate, independent cells but develop into a multicellular organism upon starvation. In this developmental programme up to 100 000 cells aggregate by chemotaxis towards cAMP. The aggregate transforms via distinct morphological states into a mature fruiting body, composed of a ball of spores supported by a thin, long stalk made of vacuolized dead cells [33]. The social amoeba is a well-established model organism for the study of several basic biological processes such as signal transduction, cell motility, cytokinesis and phagocytosis [34–37]. Despite the large evolutionary distance of around 1 billion years, many of the *D. discoideum* proteins show high sequence similarities with their human orthologues [38–40]. Thus, in recent years, the organism has become also an increasingly important model for the investigation of biological problems that are relevant to human health, such as for example autophagy, protein aggregation diseases or, as the organism can be infected with several medically relevant pathogens, host–pathogen interactions [41–43].

Here, we describe the consequences of deletion of *atg16* in AX2 wild-type and ATG9^−^ cells for development, growth, the ability to survive starvation, pinocytosis, phagocytosis and protein homoeostasis. We find that deletion of *atg16* resulted in a complex phenotype with significant defects in all of the above-stated cellular processes. Expression of GFP-tagged ATG16 largely rescued the mutant phenotype. We also find that the cell survival after starvation, and the developmental and proteasomal phenotypes of the ATG9/16 double mutant were much more severe than for either of the single mutants, while pinocytosis and phagocytosis defects were similar in all three mutant strains, suggesting that ATG9 and ATG16 probably have cellular functions in addition to their role in autophagy. Our results support links between autophagy and the uptake of nutrients and particles as well as between autophagy and the ubiquitin–proteasome system (UPS).

## 3. Results

### 3.1. Generation of ATG16^−^ and ATG16-GFP expressing strains

*Dictyostelium atg16* encodes a protein of 612 amino acids and is composed of an N-terminal half which mediates homodimerization and binding to ATG5 and a C-terminal half with seven WD40 repeats which form a β-propeller (figure 1*a*). To investigate the cellular function of ATG16, we generated *atg16* gene replacement mutants in AX2 wild-type cells and in the ATG9^−^ strain which had previously been characterized [32]. The *atg16* gene locus was inactivated by the integration of the replacement construct containing a blasticidin resistance (*bsr*) cassette (electronic supplementary material, figure S1). Successful targeted integration was confirmed by PCR of genomic DNA with gene-specific primers in combination with bsr-specific primers for two independent ATG16 knock-out strains in the AX2 background and for the *atg9/atg16* double knock-out strain. As expected, no bands were detected in the parental strains for these primer combinations (electronic supplementary material, figure S2A,B). RT-PCR of purified total cytoplasmic RNA confirmed loss of the *atg16* mRNA in the generated mutants. *atg16* cDNA of the expected size was detected in the parental strains and *pdi* (protein disulfide isomerase) cDNA as positive control was amplified in all analysed strains (electronic supplementary material, figure S2C). The two independent *atg16*^−^ strains displayed a comparable phenotype in development (data not shown). Therefore, we performed all other experiments with the ATG16^−^ #1 strain (hereafter referred to as ATG16^−^ strain). Furthermore, we generated two strains that express ATG16 C-terminally tagged with GFP in the ATG16^−^ and the ATG9^−^/ATG16^−^ background. All strains were verified by western blotting of total cell lysates and immunological detection of the corresponding proteins with specific antibodies (figure 1*b*). Table 1 [32] provides an overview of all strains used in this study.

**Table 1.** *Dictyostelium discoideum* mutant strains used in this study.

strains	summary	references
ATG9^−^	ATG9 null mutant	[32]
ATG16^−^	ATG16 null mutant	this study
ATG9^−^/16^−^	ATG9/16 double null mutant	this study
ATG16^−^/[act15]:ATG16-GFP	expression of ATG16-GFP in ATG16^−^	this study
ATG9^−^/16^−^/[act15]:ATG16-GFP	expression of ATG16-GFP in ATG9^−^/16^−^	this study
AX2/[act15]:RFP-GFP-ATG8a	expression of RFP-GFP-ATG8a in AX2	this study
ATG9^−^/[act15]:RFP-GFP-ATG8a	expression of RFP-GFP-ATG8a in ATG9^−^	this study
ATG16^−^/[act15]:RFP-GFP-ATG8a	expression of RFP-GFP-ATG8a in ATG16^−^	this study
ATG9^−^/16^−^/[act15]:RFP-GFP-ATG8a	expression of RFP-GFP-ATG8a in ATG9^−^/16^−^	this study

### 3.2. Knock-out of *atg9* or *atg16* caused similar developmental and cell survival defects which were much more severe in the double mutant

**Figure 2.**
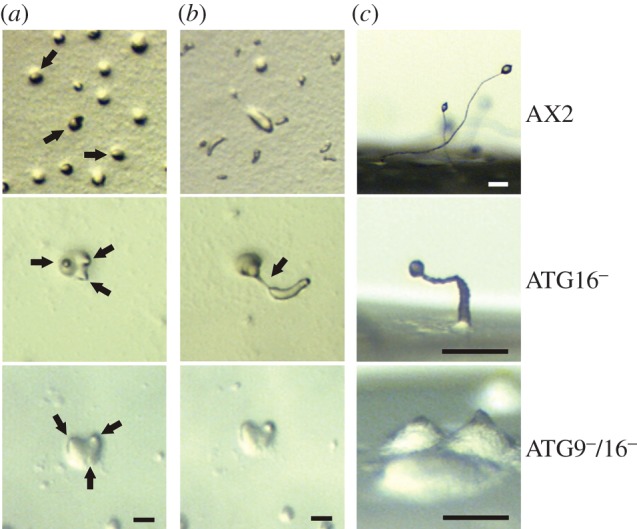
Development of the ATG16^−^ and ATG9^−^/16^−^ mutants is severely impaired. Cells were developed on phosphate agar plates and pictures were taken every 6 min. Shown are three developmental stages. (*a*) Tipped mound stage. Wild-type AX2 cells produced only one tip per mound (black arrows), while mutant cells generally produced three tips per mound (black arrows). (*b*) Slug stage. In contrast to AX2 slugs, ATG16^−^ mutant slugs were generally longer and thinner and frequently broke (black arrow). In the ATG9^−^/16^−^ double mutant, development stopped at the tipped mound stage and the mutant never proceeded to the slug stage. (*c*) Side view of terminally differentiated fruiting bodies after 48 h of development. The ATG16^−^ cells generated considerably smaller fruiting bodies (please note the different scale) that had thickened stalks in comparison to AX2 fruiting bodies. The ATG9^−^/16^−^double mutant was still arrested at the tipped mound stage. Development and generation of fruiting bodies were rescued in the ATG16^−^/ATG16-GFP strain (not shown). Scale bar, 0.1 mm.

It has been previously shown that autophagy is required for a number of developmental processes in *Dictyostelium* [32,44–46]. We investigated the development of AX2, ATG16^−^, ATG9^−^/16^−^ and the ATG16^−^/ATG16-GFP strains by starving cells on phosphate agar plates. Development was severely impaired in the ATG16^−^ mutant and the developmental phenotype was very similar to the previously reported ATG9^−^ mutant [32]. In contrast to the AX2 wild-type strain, the ATG16^−^ mutant generally generated three tips per mound in the tipped mound stage, the mutant produced longer and thinner slugs in the slug stage which frequently broke and its fruiting bodies were extremely misshapen (figure 2). The few fruiting bodies that were produced in the ATG16^−^ mutant possessed a thickened stalk and were much smaller than wild-type fruiting bodies. Re-expression of ATG16 fused C-terminally with GFP in the mutant background rescued the developmental phenotype (data not shown). The developmental defect of the ATG9^−^/16^−^ double mutant was much more severe. In contrast to the single mutants, development of the double mutant was completely arrested at the tipped mound stage and we could not detect further morphological states even after prolonged starvation of several days on phosphate agar plates (figure 2).

Next, we measured the ability of wild-type and mutant cells to survive starvation, which is more specifically reliant on autophagy than development. Cell survival was measured after starving cells in amino acid-free medium for the indicated times. We found that wild-type AX2 cells retained high viability over several days, while the survival of ATG9^−^ and ATG16^−^ strains was significantly reduced. Consistent with the most severe developmental defect, the ATG9^−^/16^−^ strain showed also the largest reduction in cell viability (electronic supplementary material, figure S3A). Thus, the ability to survive starvation confirmed the results of the developmental assay with respect to the severity of the phenotypes of the different strains.

### 3.3. Impaired autophagosome maturation in ATG9^−^, ATG16^−^ and ATG9^−^/16^−^ cells

We next assessed autophagosome number and maturation by expressing the RFP-GFP-ATG8a fusion protein in AX2 and the three mutant strains. Autophagosomes containing RFP-GFP-ATG8a emit red and green light and thus appear yellow in fluorescence microscopy, while autophagolysomes only emit red light because the fluorescence of GFP is very sensitive to the acidic environment of the lysosome. Thus, the presence of red punctae lacking the green fluorescence is an indication of the acidification of the autophagosome through fusion with the lysosome [47]. This way, the maturation of autophagosomes to autophagolysosomes can be analysed. Quantitative analysis of AX2, ATG9^−^, ATG16^−^ and ATG9^−^/16^−^ cells revealed that in AX2 cells approximately 41% of the fluorescent punctae were red indicating maturation into autophagolysosomes. In contrast, in ATG9^−^, ATG16^−^ and ATG9^−^/16^−^ cells only 8.0, 2.3 and 2.2%, respectively, of the fluorescent punctae were red (table 2; see electronic supplementary material, figure S3B, for exemplary images). This corresponds to an approximately fivefold decrease of autophagolysomes in ATG9^−^ cells, an 18-fold decrease in ATG16^−^ and a 19-fold decrease in ATG9^−^/16^−^ cells. Interestingly, the phenotype of the ATG16^−^ and ATG9^−^/16^−^ cells was comparable and much more severe than for ATG9^−^ cells. This indicates that ATG9 is less important for the completion of autophagosomal membrane expansion, which appears to be a prerequisite for fusion with the lysosome. The result is consistent with a significant reduction in autophagic flux in all three mutants which is probably caused by inefficient membrane expansion. It is also noteworthy that at least some of the large punctae in the mutant strains probably represent protein aggregates. In addition, we observed a slight decrease in the average number of autophagosomes per cell, from 1.40 for AX2 wild-type cells to 1.13 for ATG9^−^/16^−^ cells (table 2).

**Table 2.** Autophagosome maturation is impaired in ATG9^−^, ATG16^−^ and ATG9^−^/16^−^ cells.

					red punctae	
strain	cells (no.)	punctae (no.)	punctae/cell	green and red punctae (no.)	(no.)	(%)
AX2	173	242	1.40 ± 0.44	141	101	41.0 ± 9.5
ATG9^−^	143	193	1.38 ± 0.37	178	15	8.0 ± 6.2
ATG16^−^	257	323	1.24 ± 0.15	315	8	2.3 ± 1.6
ATG9^−^/16^−^	277	313	1.13 ± 0.03	306	7	2.2 ± 0.5

### 3.4. Upregulation of the autophagosomal membrane elongation machinery in ATG16^−^ cells

**Figure 3.**
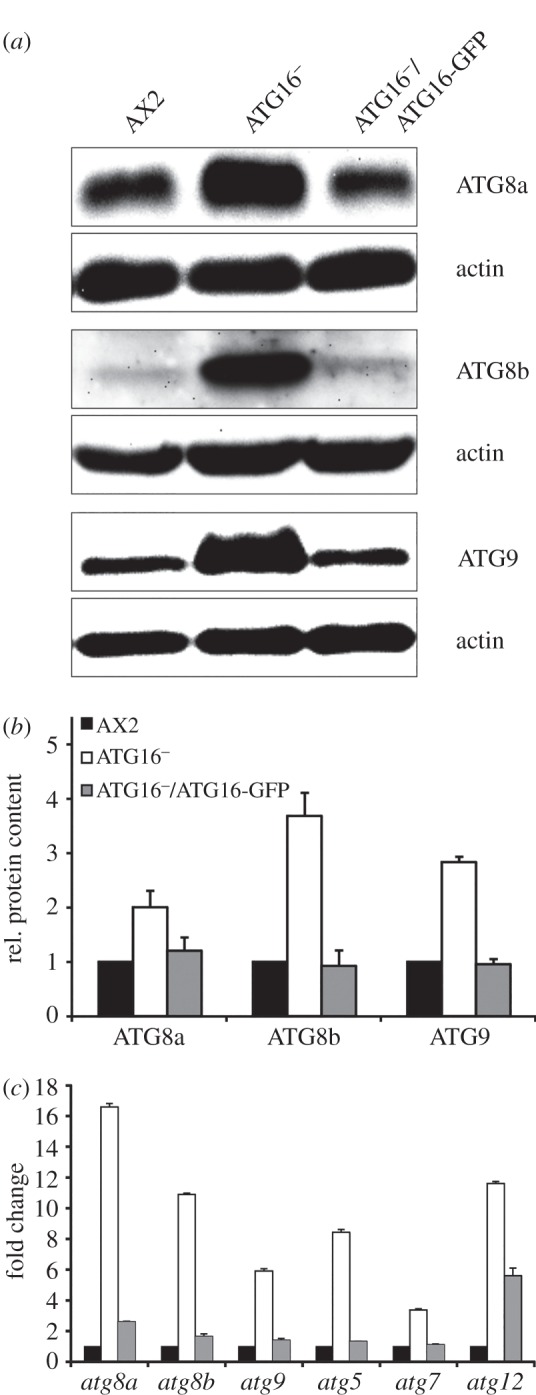
Upregulation of core autophagy genes and proteins in ATG16^−^ cells. (*a*) Representative western blots of total cell lysates of AX2, ATG16^−^ and ATG16^−^/ATG16-GFP cells. ATG8a, ATG8b and ATG9 were detected with polyclonal antibodies described previously [32,48]. Actin was used as loading control and detected with the monoclonal Act1-7 antibody. (*b*) Quantification of ATG8a, ATG8b and ATG9 protein levels in AX2, ATG16^−^ and ATG16^−^/ATG16-GFP strains. ATG8a, ATG8b and ATG9 signals were quantified by densitometric analysis and normalized based on the actin signal. Bars represent mean values and standard errors of three independent experiments. (*c*) Real-time PCR analysis of selected core autophagy genes. RNA was isolated from AX2, ATG16^−^ and ATG16^−^/ATG16-GFP cells and reverse transcribed in cDNA. Real-time PCR was carried out with primers specific for *atg8a*, *atg8b*, *atg9*, *atg5*, *atg7* and *atg12* (electronic supplementary material, table S1). Bars represent mean values and standard errors of three independent experiments.

The ATG12–ATG5/ATG16 complex acts as an E3 ligase in the lipidation of ATG8 (LC3). This is the last step in the ATG8 ubiquitin-like conjugation system and promotes the elongation of the autophagosomal membrane. *D. discoideum* encodes two ATG8 paralogues, ATG8a (DDB_G0286191) and ATG8b (DDB_G0290491), and another crucial component of the autophagosomal membrane elongation machinery is the transmembrane protein ATG9 (DDB_G0285323), which is thought to deliver membrane lipids to the growing autophagosome. To investigate whether the absence of ATG16 influences the abundance of these proteins, we analysed western blots of total cell lysates of AX2 cells, the ATG16^−^ mutant and of ATG16^−^/ATG16-GFP cells with antibodies specific for ATG8a, ATG8b or ATG9. Comparable loading of total cellular protein was confirmed by detection of actin. We observed a significant increase in protein levels for all three proteins in the ATG16^−^ strain (figure 3*a*). Quantification of three independent experiments revealed approximately twice as much ATG8a, three times as much ATG9 and four times as much ATG8b in the ATG16^−^ strain in comparison with AX2 (figure 3*b*). The levels of these proteins were approximately back to normal after expression of ATG16-GFP in the ATG16^−^ mutant (figure 3*a*,*b*). The observed increase in ATG8a, ATG8b and ATG9 protein levels could be due either to the defect in autophagosome maturation (table 2; electronic supplementary material, figure S3B), which could be responsible for their reduced degradation, or to an upregulation of gene expression. To address this issue, we performed real-time PCR analysis of the *atg8a*, *atg8b* and *atg9* genes and of *atg5*, *atg7* and *atg12* whose gene products are also part of the ubiquitin-like conjugation mechanism. The results showed a 3.5- to 17-fold increase in mRNA levels for all of these genes in the ATG16^−^ strain (figure 3*c*). Rescue of the mutant phenotype through expression of ATG16-GFP reduced the expression of *atg5*, *atg7*, *atg8b* and *atg9* to approximately AX2 wild-type levels. The mRNA levels of *atg8a* and *atg12* were also strongly reduced in the rescued strain but were still significantly higher than in AX2 wild-type cells. However, the remaining increase in transcript level of *atg8a* in the rescued strain was not reflected in the protein level (figure 3*b*,*c*). The results are consistent with the existence of a sensing system for autophagosome maturation with a positive feedback regulation of genes in this pathway.

### 3.5. ATG9 and ATG16 deficiency resulted in impaired pinocytosis and phagocytosis

**Figure 4.**
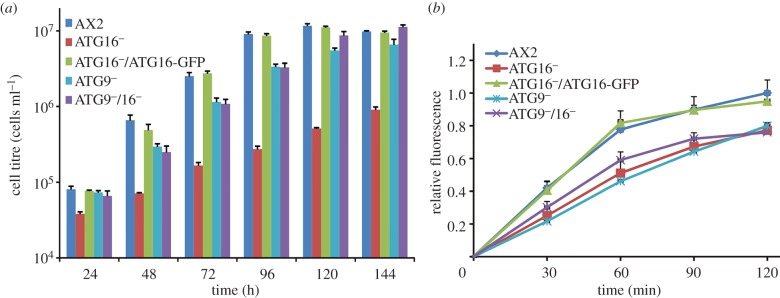
Growth in shaking culture and macropinocytosis are impaired in mutant strains. (*a*) Growth in shaking culture of AX2, ATG16^−^, ATG16^−^/ATG16-GFP, ATG9^−^ and ATG9^−^/16^−^ cells. The strains were inoculated at a starting density of 2 × 10^4^ cells ml^−1^ and the cell titre was determined every 24 h. Shown are cell titres for the first 6 days of the growth experiment. Please note the logarithmic scale of the *y*-axis. Mean values and standard errors of three independent experiments were calculated. (*b*) Macropinocytosis of TRITC-labelled dextran. The analysed *Dictyostelium* strains were adjusted to 4 × 10^6^ cells ml^−1^ and TRITC-dextran was added at a final concentration of 2 mg ml^−1^. Fluorescence with excitation at 544 nm and emission at 574 nm was determined at the indicated time points. The fluorescence of AX2 at 120 min was set to 1. Mean values and standard errors of three independent experiments were calculated.

The *D. discoideum* AX2 strain is able to grow axenically in liquid medium, a mode of growth that is supported by macropinocytic uptake of nutrients from the broth. We first analysed the growth of ATG9^−^, ATG16^−^, ATG9^−^/16^−^ and ATG16^−^/ATG16-GFP cells in comparison with AX2 in shaking culture. Log phase cells were adjusted to a density of 2 × 10^4^ cells ml^−1^ and cell proliferation was followed over 11 days in shaking culture. The ATG16^−^ strain displayed a severe growth defect over the whole time period. For example, after 72 h of growth, its cell titre was about 14 times lower than that of AX2 cells. Expression of ATG16-GFP in the ATG16^−^ strain rescued the growth defect. Unexpectedly, the growth defect of the ATG9^−^/16^−^ double mutant was similar to the ATG9^−^ strain and less severe than for the ATG16^−^ strain (figure 4*a*). Calculation of the generation times in the logarithmic growth phase revealed a dramatic increase to 22 h in the case of ATG16^−^ cells in comparison with a 12 h generation time for AX2 and the ATG16^−^/ATG16-GFP cells. The generation time of the ATG9^−^/16^−^double knock-out strain was 15 h, similar to the ATG9^−^ mutant [32]. Due to the longer generation time, the ATG9^−^ and ATG9^−^/16^−^ strains reached stationary phase between day 6 and 7, which was about 2 days later than AX2 and ATG16^−^/ATG16-GFP cells. Final cell densities were similar for these strains. The ATG16^−^ mutant reached stationary phase only after 11 days at a cell density of approximately 8 × 10^6^ cell ml^−1^, which is approximately 1.5-fold lower than the stationary cell density of AX2 (data not shown).

The growth defect of the mutant strains pointed to a pinocytosis defect and/or a defect in the intracellular utilization of nutrients. We therefore analysed the uptake of tetramethylrhodamineisothiocyanate (TRITC)-labelled dextran by AX2, ATG9^−^, ATG16^−^, ATG16^−^/ATG16-GFP and ATG9^−^/16^−^ cells. We found a significant decrease in the uptake of TRITC-labelled dextran in ATG16^−^, ATG9^−^ and ATG9^−^/16^−^ cells. The reduced pinocytosis of ATG16 deficient cells was rescued through expression of ATG16-GFP (figure 4*b*). The result suggests that autophagy has a positive function in macropinocytosis. Notably, the phenotypes of the ATG16^−^, ATG9^−^ and ATG9^−^/16^−^ strains differed with respect to growth in shaking culture and uptake of TRITC-labelled dextran. The growth defect of ATG16^−^ cells was much more severe than that of ATG9^−^ and ATG9^−^/16^−^ cells. However, the pinocytic activity of these strains was similarly reduced.

**Figure 5.**
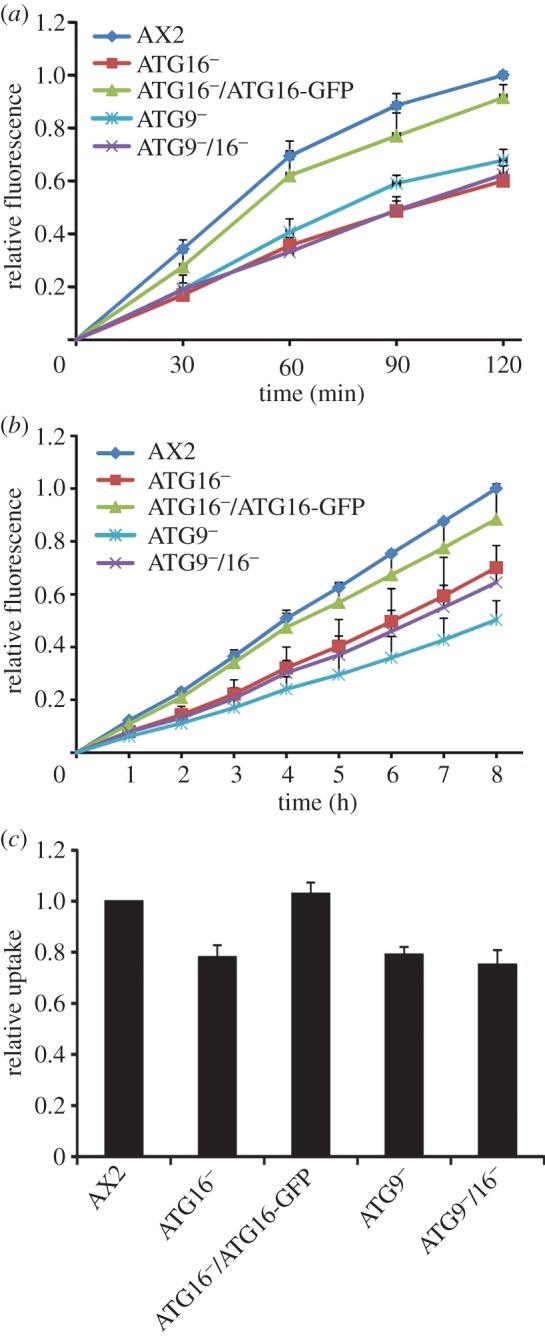
Phagocytosis is less efficient in the mutant strains. (*a*) Quantitative analysis of the phagocytosis of TRITC-labelled yeast. Cells were resuspended at 2 × 10^6^ cells ml^−1^ in Soerensen buffer, and an up to fivefold excess of fluorescent yeast was added. Data are presented as relative fluorescence and the final fluorescence of AX2 was set to 1. Mean values and standard errors of three independent experiments are shown. (*b*) Uptake of pHrodo^TM^ *E. coli*. Fluorescence of the internalized bacteria was measured at the indicated time points. Data are presented as under (*a*) and represent the mean values and standard errors of three independent experiments. (*c*) Uptake of *L. pneumophila*. Intracellular bacteria in *Dictyostelium* cells were determined 3 h post-infection with an MOI of 10. Graphs represent the mean values and standard errors of four independent experiments.

Next, we performed phagocytosis assays to further investigate the mutant strains. First, we analysed the uptake of TRITC-labelled yeast cells. AX2 cells showed a strong increase in fluorescence in the first hour of incubation with TRITC-labelled yeast. In the case of the ATG9^−^, ATG16^−^ and ATG9^−^/16^−^ cells, the increase in fluorescence was only moderate and final fluorescence values after 2 h were approximately 60% compared with AX2. Re-expression of ATG16-GFP rescued the phagocytic activity of the ATG16^−^ mutant (figure 5*a*). We then analysed the uptake of pHrodo^TM^ *Escherichia coli* particles. The fluorescence of pHrodo^TM^ *E. coli* strongly increases as the pH of its surrounding becomes acidic. This is the case when phagosomes fuse with lysosomes, therefore phagolysosomal fusion of ingested particles can be followed. We found that the fluorescence in ATG16^−^, ATG9^−^ and ATG9^−^/16^−^ strains was decreased to approximately 60% as compared with AX2 cells. ATG16^−^ cells that expressed ATG16-GFP were similar to AX2 (figure 5*b*). Both results indicate a phagocytosis defect in autophagy compromised strains. Because of the reduced pinocytotic and phagocytotic activities of the mutant strains, we next wanted to know whether the uptake of *Legionella pneumophila* would also be affected. We measured the uptake of *L. pneumophila* by determining the colony-forming units (cfu) 3 h post-infection. At this stage, intracellular bacteria have not yet started to replicate. For all mutant strains, we observed a significant decrease in the uptake of *L. pneumophila* that was approximately 75% in comparison to AX2 wild-type cells. Re-expression of ATG16-GFP rescued this defect (figure 5*c*).

### 3.6. Increase in poly-ubiquitinated proteins, appearance of protein aggregates and decrease in proteasomal activity in ATG9 and ATG16 deficient cells

**Figure 6.**
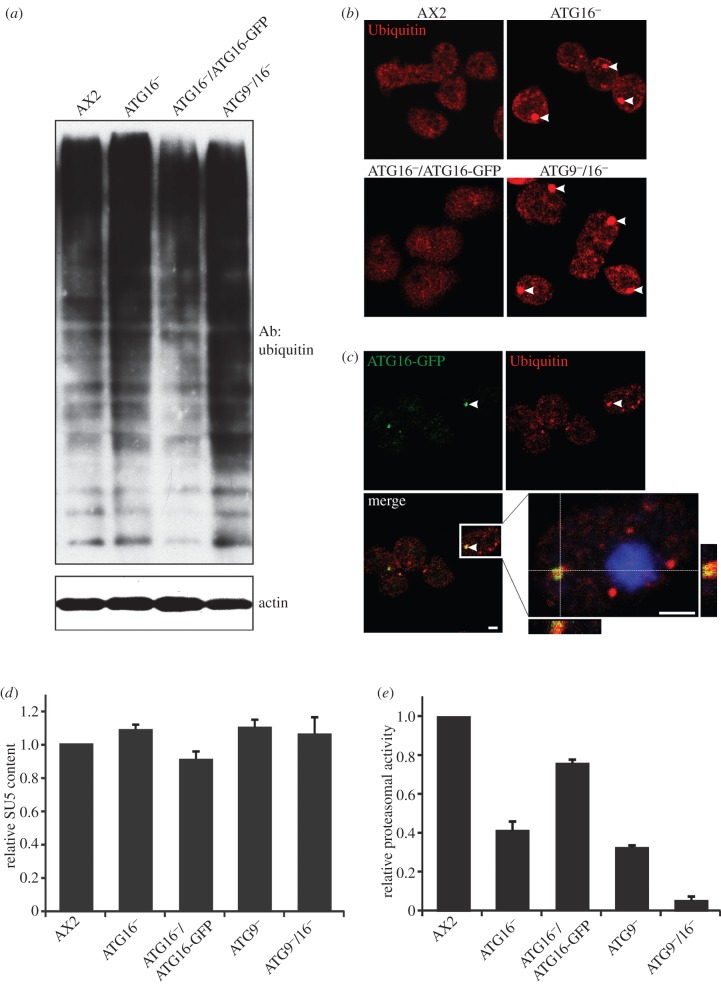
Deranged protein homoeostasis in ATG9^−^, ATG16^−^ and ATG9^−^/16^−^ cells. (*a*) Total ubiquitinylated proteins in AX2 and mutant strains. Total cell lysates of AX2, ATG16^−^, ATG16^−^/ATG16-GFP and ATG9^−^/16^−^ cells were separated by SDS-PAGE, followed by western blotting. Ubiquitinylated proteins were detected with the monoclonal P4D1 antibody against ubiquitin. Actin was used as loading control and detected with the monoclonal Act1-7 antibody. (*b*) Immunofluorescence microscopy of AX2, ATG16^−^, ATG16^−^/ATG16-GFP and ATG9^−^/16^−^ cells. Cells were fixed and stained with the P4D1 anti-ubiquitin antibody. ATG16^−^ and ATG9^−^/16^−^ cells frequently contained large ubiquitin-positive protein aggregates. (*c*) Immunofluorescence microscopy of ATG9^−^/16^−^/ATG16-GFP cells. ATG16-GFP was expressed in ATG9^−^/16^−^ double knock-out cells. Cells were fixed and stained with the anti-ubiquitin P4D1 monoclonal antibody. Nuclei are visualized by DAPI staining. In many instances and as exemplified by the enlargement of the inset, we detected partial co-localization of ATG16-GFP and ubiquitin in protein aggregates. Scale bar, 2 μm. (*d*) Expression of the proteasomal SU5 in AX2 wild-type and mutant strains. SU5 signals were quantified in AX2, ATG16^−^, ATG16^−^/ATG16-GFP, ATG9^−^ and ATG9^−^/16^−^ strains by densitometric analysis and normalized based on the actin signal. The SU5 value of AX2 has been set to 1. Bars represent mean values and standard errors of four independent experiments. A representative western blot of SU5 expression is shown in the electronic supplementary material, figure S4. (*e*) Proteasomal activity of AX2 and mutant strains. The proteasomal activity assay was performed as described in Material and methods. Proteasomal activity was normalized to proteasome content, and the chymotrypsin-like activity of AX2 was set to 1. Bars represent mean values and standard errors of three independent experiments.

The autophagolysosomal and ubiquitin–proteasome pathways are responsible for cellular homoeostasis and are the major routes for protein and organelle clearance in eukaryotic cells. They were originally considered as independent degradative pathways, however recent evidence suggests that autophagy and the UPS are interrelated [49,50]. We first analysed the presence of poly-ubiquitinated proteins in whole cell lysates of AX2, ATG16^−^, ATG16^−^/ATG16-GFP and ATG9^−^/16^−^cells using the monoclonal P4D1 antibody, which recognizes mono-ubiquitin, poly-ubiquitin and ubiquitinated proteins and cross-reacts with *Dictyostelium* ubiquitin. We detected a clear increase of ubiquitinated proteins in ATG16^−^ and ATG9^−^/16^−^cells as compared with AX2. Immunodetection of actin served as loading control (figure 6*a*). A similar increase in ubiquitinated proteins was previously observed in the ATG9^−^ strain [48]. Expression of ATG16 fused to GFP in the knock-out strain reduced ubiquitination to similar levels as in AX2 (figure 6*a*). Concomitant with the increase in poly-ubiquitinated proteins we frequently observed in immunofluorescence studies the appearance of large protein aggregates in ATG16^−^ and ATG9^−^/16^−^ cells, which stained positive for ubiquitin (figure 6*b*). Large protein aggregates positive for ubiquitin were previously also documented in ATG9^−^ cells [48]. The presence of ubiquitin-positive protein aggregates in ATG16^−^ cells prompted us to investigate a possible co-localization of ATG16 with ubiquitin. For this purpose, we expressed ATG16-GFP in the ATG9^−^/16^−^ mutant. The resulting strain showed a similar increase in protein aggregates as the ATG9^−^ mutant [48]. In confocal microscopy of fixed cells which had been stained with the P4D1 monoclonal antibody, we detected co-localization of ATG16 and ubiquitin for many protein aggregates. It appears that ATG16-GFP localized to the surface of and partially covered ubiquitin-positive aggregates (figure 6*c*).

The deficiency in autophagy in ATG9^−^, ATG16^−^ and ATG9^−^/16^−^ cells may be counteracted by the other major protein degradation pathway, the UPS. This could be achieved by an increase in proteasome number or by upregulation of proteasomal activity. Thus, we first assessed proteasome number by analysing the expression level of the proteasomal subunit 5 (SU5) in AX2, ATG16^−^, ATG16^−^/ATG16-GFP, ATG9^−^ and ATG9^−^/16^−^ cells by western blot analysis (electronic supplementary material, figure S4). Quantification of three independent experiments revealed no significant differences in the amount of SU5 in the analysed strains, indicating that expression of the proteasome is unchanged in the mutant strains (figure 6*d*). We next measured the specific proteasomal activity and normalized it to the proteasomal content. We observed a significant decrease of proteasomal activity to 40% of wild-type levels in ATG16^−^ cells and a partial rescue to about 80% in the ATG16^−^/ATG16-GFP strain. Reduction of proteasomal activity in comparison with AX2 was even higher in lysates of ATG9^−^ and ATG9^−^/16^−^ cells, where we measured 32% and 5% proteasomal activity, respectively (figure 6*e*). The further reduction of proteasomal activity in the double knock-out mutant suggests that ATG9 and ATG16 act in parallel pathways that are independently of each other important for full proteasomal activity.

## 4. Discussion

Macroautophagy is a very complex and evolutionarily highly conserved process which involves more than 20 ‘core’ ATGs, most of which have originally been described in the yeast *S. cerevisiae* [51]. Autophagosome formation is governed by the ULK1 complex, the PI3K complex, two ubiquitin-like protein conjugation systems and several additional proteins. ATG16 and ATG8 (LC3) are central components of the two ubiquitin-like protein conjugation systems, which include eight core ATG proteins and are crucial for the expansion of the membrane of the growing PAS in yeast or the phagophore in, for example, mammals and *Dictyostelium* [3]. ATG16 associates non-covalently with the ATG12–ATG5 conjugate and forms a tetrameric complex which catalyses ATG8 lipidation as an E3 ligase [12,13]. In *S. cerevisiae*, the ATG12–ATG5/ATG16 complex resides on the PAS, but is no longer present on the completed autophagosome [14]. Similarly, ATG12–ATG5/ATG16L1 predominantly localizes on the outer surface of the phagophore in mammals and dissociates from the membrane immediately before or after completion of the autophagosome [7]. Thus, it is assumed that the ATG16 complex delivers ATG8 through lipidation to the membrane of the forming autophagosome and that ATG8 then functions in the closing of the autophagosome [11]. Another important player in autophagosomal membrane elongation is the transmembrane protein ATG9, which is thought to deliver membrane lipids [52–54]. We reasoned that the abundance of ATG proteins involved in membrane expansion must be coordinately regulated and that deletion of an essential protein in this process such as ATG16 could influence the concentration of the other proteins. Indeed, we found that the protein levels of ATG8a, ATG8b and ATG9 were strongly increased in the ATG16 knock-out strain. The accumulation of ATG8a and ATG8b could be caused by autophagy deficiency in the ATG16 mutant. As autophagosomes cannot be completed in this mutant, a subfraction of ATG8a and ATG8b would no longer be degraded and hence would accumulate. However, the observed block in autophagosome maturation in the ATG16 mutant (table 2) cannot satisfactorily explain the increased level of ATG9 because it has been shown that ATG9 is recycled upon autophagosome completion [55]. An alternative explanation for the strong upreglation of ATG8a, ATG8b and ATG9 in the ATG16 mutant would be a sensing system for autophagosome completion which would regulate expression of the involved proteins. Therefore, we analysed the transcription of these genes and of *atg5*, *atg7* and *atg12*, whose gene products are also important for autophagosomal maturation, by real-time PCR. The results showed that the expression of all of these genes was significantly increased in the ATG16 knock-out strain. This indicates that the increase in protein levels of ATG8a, ATG8b and ATG9 was mainly caused by an upregulation of their expression in ATG16^−^ cells. We predict that also *atg3*, *atg4* and *atg10*, as being also part of the two ubiquitin-like protein conjugation systems which are essential for elongation of the autophagosomal membrane, are upregulated in ATG16^−^ cells. How the cell senses the status of the autophagosome and elicits the corresponding response needs to be addressed in future work.

It is well established that autophagy plays an important role in differentiation and development in multicellular eukaryotes [56]. The same is true for *Dictyostelium,* and previous studies showed that deletion of *atg1*, *atg5*, *atg6*, *atg7*, *atg8*, *atg9* or *vmp1* caused aberrant development of varying severity [32,44–46]. We found that also deletion of *atg16* resulted in a strong developmental phenotype which was very similar to the ATG9^−^ strain. Loss of either protein led to severe impairments in the tipped aggregate stage, the slug stage and in fruiting body generation (figure 2) [32]. The observed phenotypes were also similar to the ones reported for the knock-out mutants of ATG5 and ATG7 [45]. The similar developmental phenotypes for these knock-out strains make perfect sense, because all four proteins are essential components of the two ubiquitin-like protein conjugation systems involved in the elongation of the autophagosomal membrane. The developmental phenotype of the ATG9/16 double knock-out mutant was therefore of considerable interest. Unexpectedly, loss of both proteins led to a much more severe phenotype with an early developmental arrest at the tipped mound stage. As both proteins function in membrane elongation of the phagophore, we assessed autophagosome maturation in the mutant strains by monitoring RFP-GFP-ATG8a (table 2; electronic supplementary material, figure S3B). We found that autophagosome maturation was similarly impaired in ATG16^−^ and ATG9^−^/16^−^ cells, but to a lesser extent in ATG9^−^ cells. Next, we measured the ability of the cells to survive starvation, which is more specifically reliant on autophagy, and found a similar decrease in survival in the single knock-out mutants, while the ATG9^−^/16^−^ cells were much more severely impaired (electronic supplementary material, figure S3A). The results suggest that there is still some albeit strongly reduced autophagic activity in the single mutants which is sufficient for development to proceed beyond the tipped mound stage. The much more severe developmental phenotype of the double mutant in conjunction with a similar deficiency in the ability to survive starvation suggests that ATG9 and ATG16 act in parallel in autophagosome formation.

The ATG9^−^ and ATG9^−^/16^−^ mutants displayed also defects in pinocytosis, as evidenced by the reduced uptake of TRITC-labelled dextran, and phagocytosis, as evidenced by phagocytosis of TRITC-labelled yeast, pHrodo™ *E. coli* BioParticles® and the uptake of the clinically relevant human pathogen *L. pneumophila* (figures 4 and 5). The link between autophagy and phagocytosis is not clear. One possibility is that disruption of cellular recycling processes may lead to a shortage of membranes and other components that are needed for efficient phagocytosis. Alternatively, there may be a direct link between autophagy and phagocytosis. The defect in pinocytosis might be explained by the fact that ATG9 and ATG16 are localized to recycling endosomes and may have important functions in endocytosis [53]. Thus, knock-out of either protein could lead to the observed pinocytic defect (figure 4).

The complex phenotypes of the ATG16^−^ and ATG9^−^/16^−^ strains were similar to the ATG9 knock-out strain with the exceptions of growth in shaking culture, survival upon starvation, development and proteasomal activity. This suggests that both proteins may, at least partially, act in parallel in these cellular processes. Surprisingly, the growth phenotype of the ATG9/16 double mutant was similar to the ATG9^−^ strain and milder than for the ATG16^−^ strain, while development and proteasomal activity were much more severly impaired in the ATG9^−^/16^−^ strain (figures 2, 4 and 6). We currently do not have an explanation for this opposing result. The strong decrease in proteasomal activity in the ATG9^−^, ATG16^−^ and ATG9^−^/16^−^ strains was mirrored by an increase in total poly-ubiquitinated proteins and the appearance of large protein aggregates which stained positive for ubiquitin (figure 6). Autophagy and the UPS are the two major intracellular pathways for protein degradation and are crucial for normal protein homoeostasis. Until recently, they were thought to act independently, but evidence is accumulating that there is crosstalk between the two pathways [49,57,58]. It is well established that there is compensatory upregulation of autophagy upon inhibition of proteasomal activity [49,59–61]. The picture with respect to a possible compensatory regulation of the UPS in the case of autophagy deficiency is less clear. Wang *et al*. [62] recently reported an upregulation of proteasomal subunits and an increase in proteasomal activity upon pharmacological inhibition of autophagy and also upon downregulation of autophagy genes by RNA_i_ in colon cancer cells. By contrast, autophagy inhibition through treatment with the autophagy inhibitor bafilomycin or siRNA knock-down of *atg7* or *atg12* in HeLa cells resulted in impaired clearance of UPS clients. The authors concluded that autophagy inhibition may impact the flux through the UPS and that this deficiency is predominantly mediated by the adaptor molecule p62/SQSTM1 [63]. Of note, also a decrease of proteasomal activity in the case of autophagy reduction has been observed. Inhibition of lysosomal activities by chloroquine in neuroblastoma cells resulted in an accumulation of ubiquitinated proteins and reduced proteasomal activities. Furthermore, in mice deficient for the lysosomal enzyme cathepsin D proteasomal activities were also significantly reduced [64,65]. We found an increase of poly-ubiquitinated proteins and the appearance of large ubiquitin-positive protein aggregates in ATG9^−^, ATG16^−^ and ATG9^−^/16^−^ cells, which indicated a strong imbalance in protein homoeostasis in these cells. We hypothesized that the disturbance of protein degradation might be compensated through either an upregulation of the proteasome number or proteasomal activity. However, we found no significant difference in the expression level of proteasomal SU5 in these strains and, to our surprise, proteasomal activity of ATG9^−^ and ATG16^−^ cells was strongly reduced and the proteasomal activity of the double mutant was even more compromised (figure 6*e*; electronic supplementary material, figure S4). This result clearly shows that, opposite to the expectation, there is no compensatory increase of the UPS in these strains. Accordingly, an intact autophagy pathway is needed for full proteasomal activity in *Dictyostelium* cells. The further significant reduction of proteasomal activity in the ATG9^−^/16^−^ strain suggests that both proteins, ATG9 and ATG16, have overlapping as well as independent functions that are needed for full proteasomal activity. Thus, it appears that at least some human or mouse cell types as well as *Dictyostelium* cannot compensate an imbalance in protein degradation because of an autophagic block through an upregulation of the UPS. Rather, the imbalance is exacerbated because the second arm of protein degradation gets also severly compromised.

## 5. Conclusion

In summary, our results clearly demonstrate a crosstalk between autophagy and the UPS with the unexpected outcome that a functioning autophagy is essential for optimal proteasomal activity. They also suggest that the core ATGs, ATG9 and ATG16, have likely additional autophagy-independent functions. This could explain why the phenotypes of the ATG9^−^, ATG16^−^ and ATG9^−^/16^−^ strains are similar for some cellular processes but different for others.

## 6. Material and methods

### 6.1. *Dictyostelium* strains, growth and development

*Dictyostelium discoideum* AX2 was used as wild-type strain. The ATG16^−^ and ATG9^−^/16^−^ strains were generated by replacing part of the *atg16* gene with the Bsr resistance cassette in AX2 and ATG9^−^ cells. ATG16^−^/ATG16-GFP and ATG9^−^/16^−^/ATG16-GFP mutants were generated by transformation of ATG16^−^ and ATG9^−^/16^−^ cells, respectively, after transient expression of the Cre recombinase with an expression construct encoding GFP fused C-terminally to ATG16. Strains used in this study are listed in table 1. AX2 and mutant cells were grown at 21°C in Ax2 medium (for 1 l: 14.3 g bacteriological peptone, 7.15 g yeast extract, 18 g maltose, 0.62 g Na_2_HPO_2_ × 2H_2_O, 0.49 g KH_2_PO_4_, pH 6.7) that contained in the case of mutant strains in addition 5 µg ml^−1^ Blasticidin S (ICN Biochemicals) or 6 µg ml^−1^ G418 (Sigma) with shaking at 160 r.p.m. [66] or on SM agar plates with *Klebsiella aerogenes* [67]. For growth experiments, log phase cells with a cell titre of 2–4 × 10^6^ cells ml^−1^ were inoculated at a density of 2 × 10^4^ cells ml^−1^ in 30 ml Ax2 medium and growth was monitored by measuring the cell titre every 24 h. For the calculation of generation times in the logarithmic growth phase, cell titres of AX2 and the ATG16^−^/ATG16-GFP strain between days 2 and 3, of ATG9^−^/16^−^ and ATG9^−^ between days 3 and 4 and of ATG16^−^ between days 6 and 8 were used. Generation times were calculated using *n* = [log*N_t_* − log*N*_0_]/log2 where *n* is the number of generations, *N*_0_ is the cell titre at time 0 and *N_t_* is the cell titre at time *t*. For the measurement of cell survival upon starvation, cells were resuspended in SIH medium without amino acids (Formedium, UK) and cell survival was determined at the indicated times by counting the number of cfu on lawns of *K. aerogenes*. Two independent experiments with two parallel cultures each were performed, day 1 was set to 1 for each strain and standard errors of the mean were calculated.

For monitoring development on phosphate agar plates, log phase cells from a shaking culture were washed twice with Soerensen buffer (2.0 mM Na_2_PO_4_, 14.6 mM KH_2_PO_4_, pH 6.0). A total of 1 × 10^8^ cells were then resuspended in 1 ml of Soerensen buffer and 500 µl of this solution corresponding to 5 × 10^7^ cells was evenly distributed onto a phosphate-buffered agar plate (9 cm in diameter) and incubated at 21°C. Different stages of development were observed and the images were captured either manually at defined time points or automatically at 6 min intervals using a stereomicroscope (MZFIII, Leica, Germany).

### 6.2. Vector construction and transformation

The *atg16* gene replacement construct was generated in the pLPBLP vector where the Bsr resistance cassette is flanked by loxP sites [68]. A PCR-amplified fragment of 521 bp (bases 23–543 of the coding sequence) was cloned into the *Bam*HI and *Pst*I sites and a 646 bp fragment (bases 1630–2276) into the *Hind*III and *Sal*I sites of the pLPBLP vector. The plasmid was introduced into AX2 cells and the ATG9^−^ mutant by electroporation, and transformants were selected in the presence of 5 μg ml^−1^ Blasticidin S. Gene replacement mutants were identified by PCR screening of blasticidin resistant clones followed by western blotting. For ectopic expression of full-length ATG16 fused N-terminally to GFP, the pBsr-C-GFP C1 vector was used [69]. The full-length ATG16-coding sequence was amplified by PCR and cloned into the pBsr-C-GFP C1 vector via the *Bam*HI and *Xma*I restriction sites, and the final expression construct was verified by sequencing. In the ATG16-GFP fusion protein, a linker of six amino acids with the sequence GGSGGS, which was introduced through the PCR primer, separates the GFP moiety from the full-length ATG16. The plasmid was introduced into ATG16^−^ and ATG9^−^/16^−^ mutants by electroporation and transformants were selected in the presence of 5 µg ml^−1^ Blasticidin S. Blasticidin resistant clones expressing ATG16-GFP were identified by visual inspection under a fluorescence microscope and verified by western blotting.

### 6.3. Antibody generation, SDS-PAGE, western blotting and protein quantification

For generation of ATG16-specific polyclonal antibodies (pAbs), the cDNA sequence encoding the coiled-coil domain (aa 160–210) was amplified by PCR and cloned into the pGEX-6p-1 GST expression vector. The fusion protein was expressed in *E. coli* XL1 blue, purified using GST-Sepharose beads, released through cleavage with PreScission protease and used for the immunization of rabbits (BioGenes GmbH, Germany). SDS-PAGE and western blotting were essentially performed as described [70,71]. The proteins of 2 × 10^5^ cells per lane were separated by SDS gel electrophoresis of total cell lysates. The generated ATG16 pAbs were affinity purified and used for western blotting at a 1 : 1000 dilution. GFP was detected with monoclonal antibody K3-184-2 at a 1 : 15 dilution [72]. The proteasomal SU5 was detected with monoclonal antibody 171-337-2 at a 1 : 100 dilution [73]. Actin was detected with monoclonal antibody Act1-7 at a 1 : 15 dilution [74]. ATG9, ATG8a and ATG8b were detected with specific pAbs at a 1 : 10 000 dilution as described [32,48]. Secondary antibodies used were anti-mouse and anti-rabbit lgG conjugated with peroxidase (Sigma) followed by chemiluminescence detection. Images were recorded and analysed using the Fluorchem SP imaging system (Alpha Innotech, USA). Relative protein amounts were determined densitometrically using the Spot Denso tool of the AlphaEaseFC software (Alpha Innotech). Background values were subtracted and the resulting intensities normalized based on actin staining. Mean values and standard errors of three independent experiments were calculated.

### 6.4. Phagocytosis and pinocytosis analysis

Quantitative phagocytosis of TRITC-labelled heat-killed yeast cells was performed as described [75]. Briefly, *D. discoideum* cells at a density of 2–4 × 10^6^ cells ml^−1^ were washed twice with Soerensen buffer and resuspended at a density of 2 × 10^6^ cells ml^−1^ in Soerensen buffer. One millilitre of the cell suspension was centrifuged for 4 min at 500*g* in an Eppendorf tube, the supernatant was carefully removed and the pellet frozen at −20°C. This sample was used for the determination of the protein content. Ten microlitres of the cell suspension was dispensed in a 30-ml Erlenmeyer flask and incubated for 15 min on a rotary shaker to allow cells to recuperate. Then TRITC-labelled yeast cells were added at a fivefold excess relative to *Dictyostelium* cells. A 1-ml sample was removed immediately (time point 0) and transferred to an Eppendorf tube containing 100 μl 0.4% Trypan Blue solution to quench external fluorescence, washed twice with Soerensen buffer, the cell pellet resuspended in 500 µl Soerensen buffer and fluorescence measured immediately using an Infinite M1000 plate reader (Tecan Group Ltd., Switzerland) at 544 nm excitation and 574 nm emission. The other time points (30, 60, 90 and 120 min) were processed as described for the 0 time point. The measured fluorescence values were corrected with the background fluorescence of cells at time point 0 and for differences in protein content. Mean values and standard errors of three independent experiments were calculated. Quantitative phagocytosis of pHrodo^TM^ *E.coli* Bioparticles (Invitrogen) was performed as described [32]. Mean values and standard errors of four independent experiments were calculated. The uptake of *L. pneumophila* Corby by *Dictyostelium* AX2 cells and mutant strains was measured after 3 h of infection. The infection assay and the determination of intracellular bacteria were performed as described [32].

Fluid phase macropinocytosis was determined using TRITC-labelled dextran [76]. *Dictyostelium* cells at a density of 2–4 × 10^6^ cells ml^−1^ were pelleted and resuspended at 4 × 10^6^ cells ml^−1^ in fresh nutrient medium. One millilitre of the cell suspension was centrifuged for 4 min at 500*g* in an Eppendorf tube, the supernatant was carefully removed and the pellet was frozen at −20°C. This sample was used for the determination of the protein content. Five millilitres of the cell suspension was dispensed in a 30-ml Erlenmeyer flask and incubated for 15 min on a rotary shaker to allow cells to recuperate. Then TRITC-labelled dextran (Sigma) was added to a final concentration of 2 mg ml^−1^. Five hundred microlitre samples were removed immediately (time point 0) and after 30, 60, 90 and 120 min and processed and fluorescence measured as described for the phagocytosis of TRITC-labelled yeast cells (see above). The measured fluorescence values were corrected by the background fluorescence of cells at time point 0 and for differences in protein content. Mean values and standard errors of three independent experiments were calculated.

### 6.5. Fluorescence microscopy

Immunofluorescence microscopy was essentially done as described [32]. Fixed cells were incubated with the monoclonal P4D1 anti-ubiquitin antibody (Cell Signaling Technology) at a 1 : 100 dilution in PTB (1× PBS, 0.1% Triton X-100, 0.1% BSA). Secondary antibody was Alexa-fluor 568 conjugated goat anti-mouse IgG at a 1 : 2000 dilution (Invitrogen). Nuclei were stained with 4′,6-diamidino-2-phenylindole (DAPI, Sigma). Confocal images of fixed cells were recorded in sequential mode with an inverted TCS SP5 laser scanning microscope (Leica) with a 100× HCX PL APO NA 1.40 oil immersion objective. The RFP-GFP-ATG8a autophagosome maturation assay was essentially done as described [47]. Briefly, incorporation of RFP-GFP-ATG8a into the autophagolysosome can be monitored because the acidic environment of the lysosome quenches the more sensitive fluorescence of GFP while the fluorescence of RFP is preserved for longer. Therefore, yellow punctae (green and red) indicate autophagosomes, while red punctae (green fluorescence is quenched) indicate autophagolysosomes. To allow monitoring of RFP-positive punctae, lysosomal degradation was slowed down by treatment of the cells with 100 mM NH_4_Cl twice for 2 h. *In vivo* imaging was done with an inverted TCS SP5 laser scanning microscope (Leica). Three independent experiments were performed and the number of RFP-GFP-ATG8a punctae were counted from single plane. Excitation of GFP was at 488 nm, emission 500–550 nm; of Alexa-fluor 568 at 568 nm, emission 600–630 nm; and of DAPI at 405 nm, emission 412–464 nm. Images were processed using the accompanying Leica Application Suite (LAS) software.

### 6.6. Proteasomal activity analysis

Proteasomal activity assays of the different *D. discoideum* strains were performed using the established protocol from skeletal muscle tissue [77] with minor changes as described [48]. Mean values and standard errors of three independent experiments were calculated. The chymotrypsin-like activity of AX2 wild-type cells was set to 1.

### 6.7. Miscellaneous methods

RNA isolation, cDNA generation and real-time PCR were essentially done as described [78]. Table S1 of the electronic supplementary material lists the primers that were used in this study.

## Supplementary Material

Strategy for the generation of the atg16 gene replacement mutant in AX2 and ATG9^−^ cells.

## Supplementary Material

Verification of the generated ATG16^−^ and ATG9^−^/16^−^ knock-out strains

## Supplementary Material

Cell survival upon starvation and autophagosome maturation is impaired in ATG9^−^, ATG16^−^ and ATG9^−^/16^−^ cells

## Supplementary Material

Analysis of proteasomal subunit 5 (SU5) expression
