# Supplementary figures and images for: The phenotypes of ATG9, ATG16 and ATG9/16 knock-out mutants imply autophagy-dependent and -independent functions
Source: Open Biol. 2015 Apr 15;5(4):150008. doi: 10.1098/rsob.150008 (PMC4422124; doi:10.1098/rsob.150008)

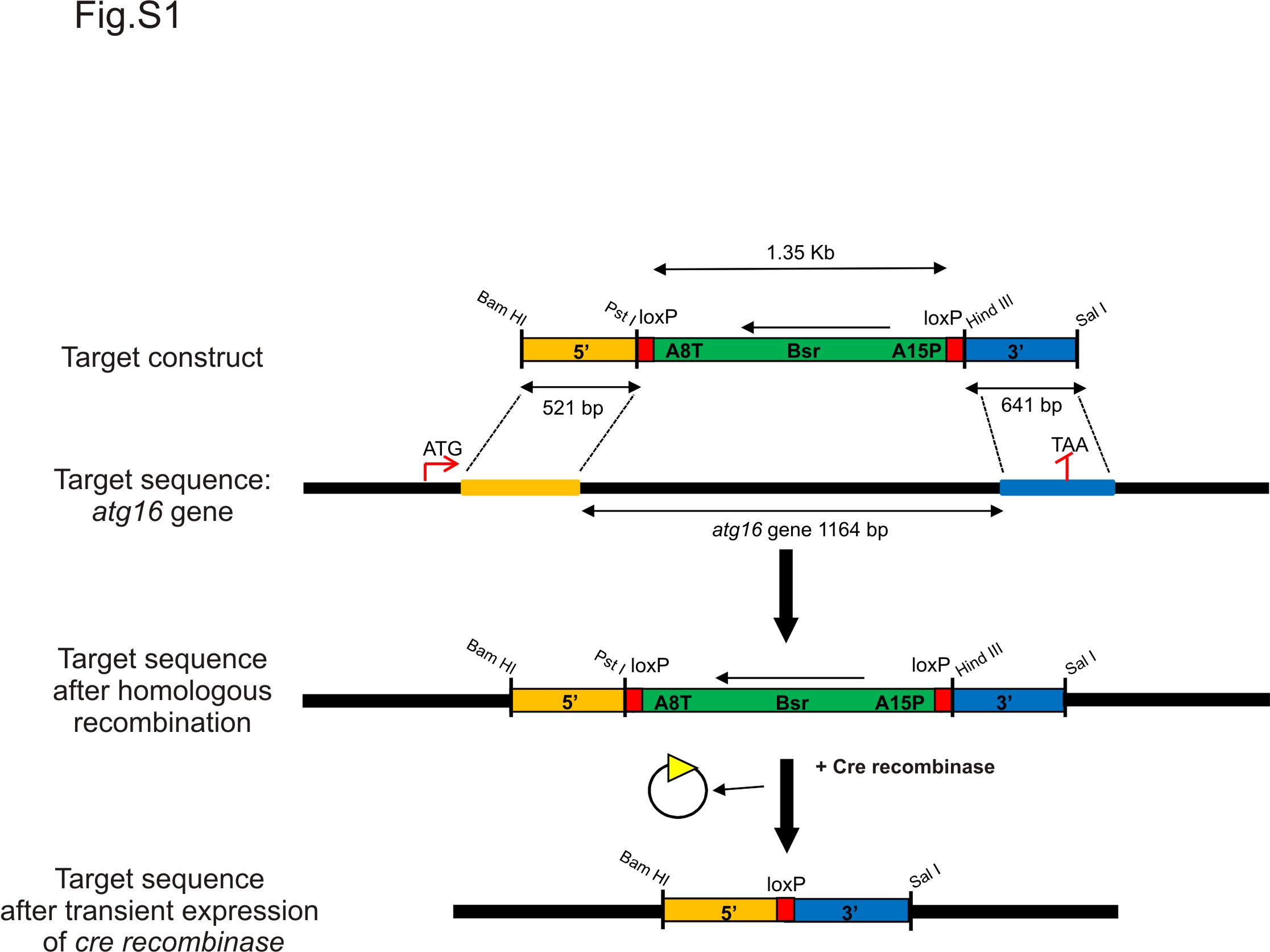

Supplement: Strategy for the generation of the atg16 gene replacement mutant in AX2 and ATG9− cells. [file rsob150008supp1.jpg]

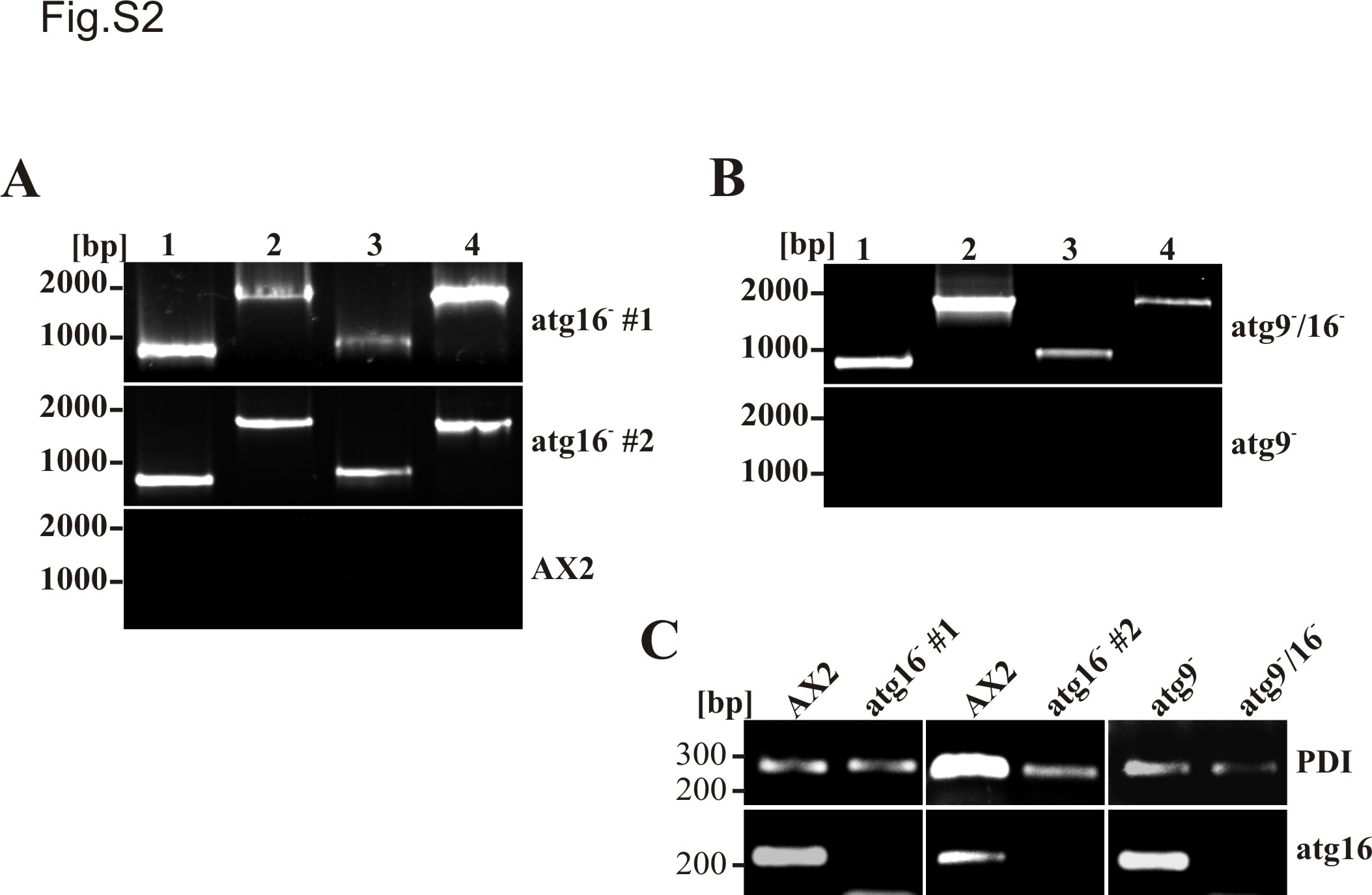

Supplement: Verification of the generated ATG16− and ATG9−/16− knock-out strains [file rsob150008supp2.jpg]

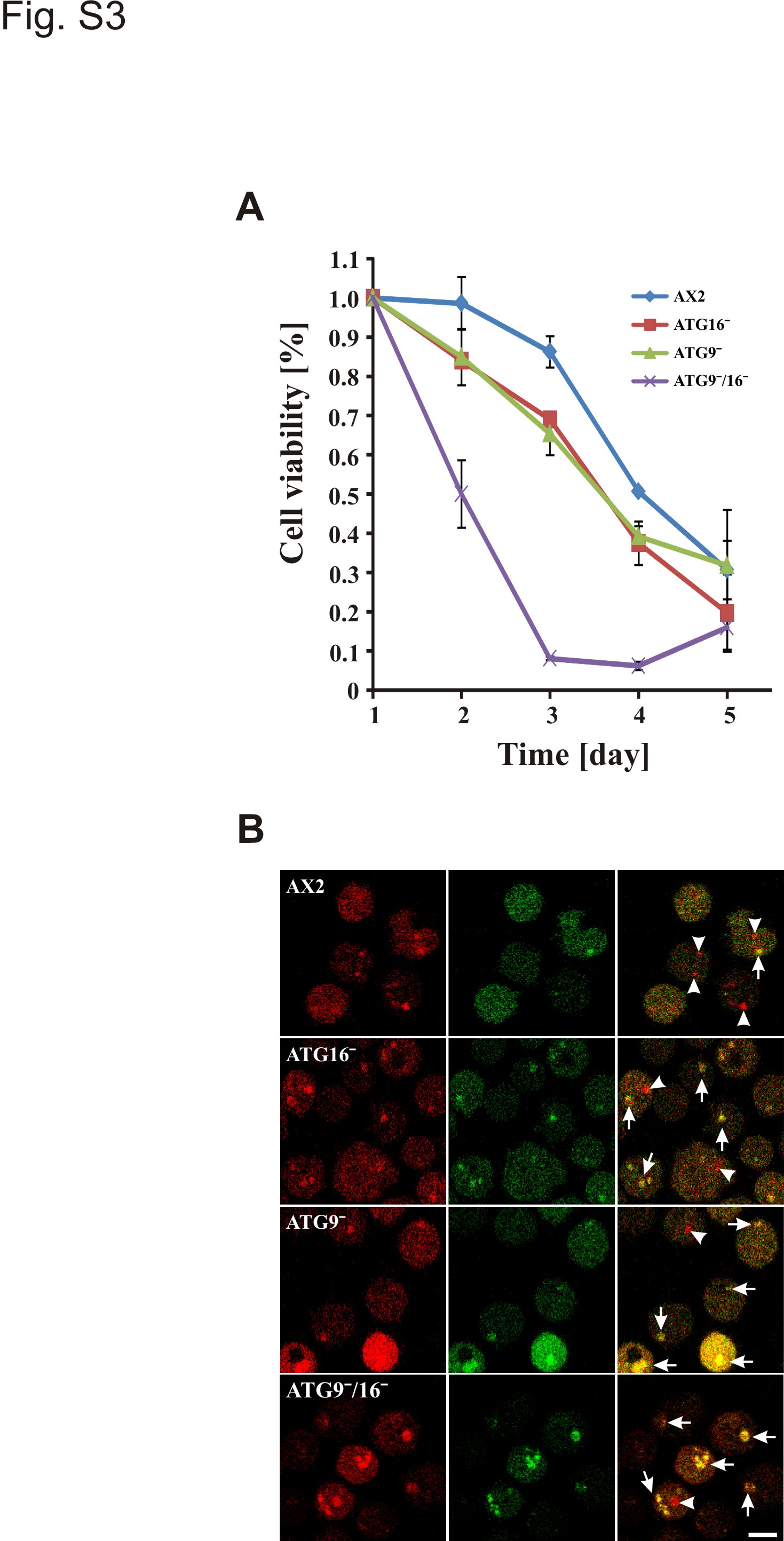

Supplement: Cell survival upon starvation and autophagosome maturation is impaired in ATG9−, ATG16− and ATG9−/16− cells [file rsob150008supp3.jpg]

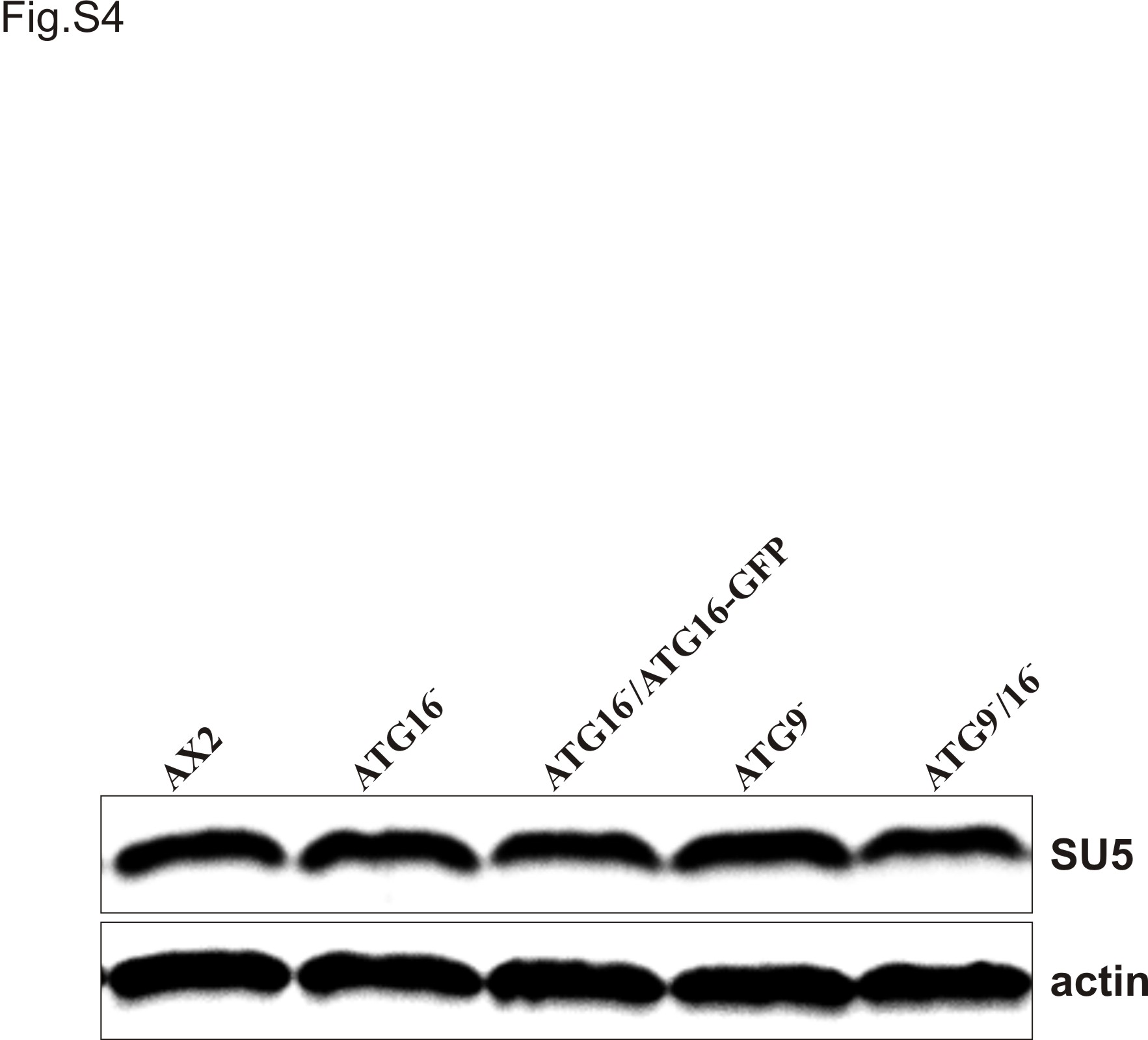

Supplement: Analysis of proteasomal subunit 5 (SU5) expression [file rsob150008supp4.jpg]
